# Laparoscopic Radical Trachelectomy after Neoadjuvant Chemotherapy for Fertility Preservation in Early-Stage Bulky Cervical Cancer: A Case Report and Literature Review

**DOI:** 10.3390/medicina58121827

**Published:** 2022-12-12

**Authors:** Danuta Vasilevska, Andrzej Semczuk, Dominika Vasilevska, Artiomas Širvys, Vilius Rudaitis

**Affiliations:** 1 Department of Gynecology, Vilnius University Hospital Santaros Clinics, 08661 Vilnius, Lithuania; 2 2nd Department of Gynecology, Lublin Medical University, 20090 Lublin, Poland; 3 Faculty of Medicine, Vilnius University, 03101 Vilnius, Lithuania

**Keywords:** bulky early-stage cervical cancer, fertility-sparing treatment, laparoscopic radical trachelectomy, neoadjuvant chemotherapy

## Abstract

Management of early-stage cervical cancer (CC) in young women often faces challenges to preserve fertility, as well as to achieve an adequate oncological outcome. Although existing evidence supports a fertility-sparing treatment in the case of tumors <2 cm in diameter, the approach is less clear in bulky early-stage CC. In addition, the outcomes of radical trachelectomy performed by minimally invasive techniques are also highly debatable. Highlighting the high incidences of young women with early-stage CC, the lack of sufficient data raises considerable hindrances towards the proper counseling of this vulnerable patient group. In this report, a case of a young woman with bulky early-stage CC with a strong desire to preserve fertility is presented. A satisfactory oncological outcome was achieved after neoadjuvant chemotherapy followed by laparoscopic radical trachelectomy. Ongoing prospective trials are expected to provide stronger evidence on this topic.

## 1. Introduction

Cervical cancer (CC) is ranked as the fourth most common oncological disease among women, with a peak incidence in the younger population—between 35 and 45 years of age [1]. Historically, widely accepted treatment for early-stage CC was considered a radical hysterectomy (RH) with pelvic lymph nodes dissection. Consequently, developed fertility-sparing surgical (FSS) techniques, including trachelectomy and cone biopsy, were used from the 1990s in cases when childbearing was strongly desired. Fertility-sparing treatment is performed in the early-stage CCs, especially in the stages IA1, IA2, and IB1 with tumors below 2 cm in diameter, with the absence of lymph node metastases and perineural spread. The approaches of radical trachelectomy include vaginal, abdominal, or minimally invasive. Moreover, it is usually accompanied by pelvic lymph node dissection and exclusively neoadjuvant chemotherapy [2]. Fertility-sparing treatment offers similar oncologic outcomes, compared to traditional treatment, in thoroughly selected patients, though it is associated with certain obstetrics complications [3]. Fertility-sparing CC treatment carries certain ethical dilemmas, highlighting the importance of adequate counseling of the patient and respecting their opinions and beliefs. Since there is still a lack of strong recommendations on the best overall approach of radical trachelectomy, the choice is usually based on local resources, as well as preferences of the surgeon [4]. The aim of the current case report was to provide an outcome of the early-stage CC woman who had a strong desire to preserve her fertility. She was treated with neoadjuvant chemotherapy and laparoscopic radical trachelectomy, achieving a satisfactory oncological outcome.

## 2. Case Report

**Figure 1:**
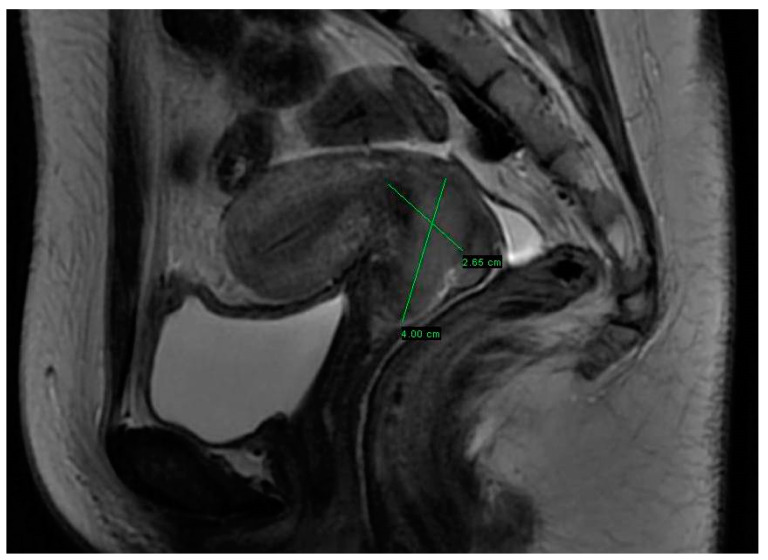
Pelvic MRI performed at the initial evaluation of the patient, revealing cervical tumor 40 mm in the longest diameter.

A 25-year-old patient, with nulligravida and body mass index 23.5 kg/m^2^, had undergone the first Pap smear screening, which revealed atypical squamous cells of undetermined significance (ASC-US). After the repeated Pap smear, 6 months later, a high-grade squamous intraepithelial lesion (HSIL) with human papillomavirus (HPV) type 16 infection was diagnosed. The specimen collected during uterine cervix biopsy showed squamous G3 cervical carcinoma. To determine the spread of the disease, pelvic magnetic resonance imaging (MRI) was immediately carried out, showing cervical tumor, 40 mm in the longest diameter, without clear parametrial invasion; on the left side, pathological obturator lymph node 15 × 10 mm in size was noticed, while obturator lymph nodes on the right side were up to 8 mm in diameter (Figure 1). For more accurate oncological diagnosis, whole body computerized tomography (CT) was performed, revealing 39 × 20 mm posterior cervical mass and internal iliac lymph nodes on the left, up to 12 mm in diameter, and on the right, up to 10 mm, with no disease spread in the abdomen. The lymph nodes were highly suspicious for cervical cancer metastasis. Altogether, after clinical and radiological evaluations, cervical cancer cT2bN1M0G3, FIGO stage IB2, was diagnosed. Video S1 shows the colposcopic image of uterine cervix during initial evaluation.

After diagnosis, indocyanine green (ICG)-enhanced fluorescence-guided laparoscopy was carried out, performing paraaortic and obturator lymphadenectomy. The ICG solution was prepared with 25 mg of ICG powder diluted into 10 mL of sterile aqueous water. Afterwards, the cervix was injected with 2 mL of ICG at the 3, 6, 9, and 12 o’clock positions, with 1 mL superficially under the mucosa and 1 mL 1 cm into the cervical stroma. Moreover, the bilateral salpingectomy and ovarian transposition was done. As discussed, the treatment plan with patient after first radiologic staging and interdisciplinary counselling, ovarian transposition, and radical radiotherapy was suggested, due to the bulky CC and suspicion of pelvic lymph nodes metastases. Liver, peritoneum, omentum, and other organs were without visible pathological changes. The uterus was normal in size, and a decent amount of pelvic free fluid was observed in the Douglas pouch. Pelvic examination under infrared light showed ICG dye distribution in pelvic and paraaortic lymph nodes.

Removal of obturator and paraaortic lymph nodes were performed. Surgical procedure is shown in Video S2. No frozen section of lymph nodes was performed. Histopathological evaluation unexpectedly revealed the reactive lymphadenopathy in all resected lymph nodes (two right iliac, four left iliac, and two paraaortic lymph nodes). The patient was believed to have positive lymph nodes and be a candidate for radiotherapy; hence, only selective lymphadenectomy was performed.

**Figure 2:**
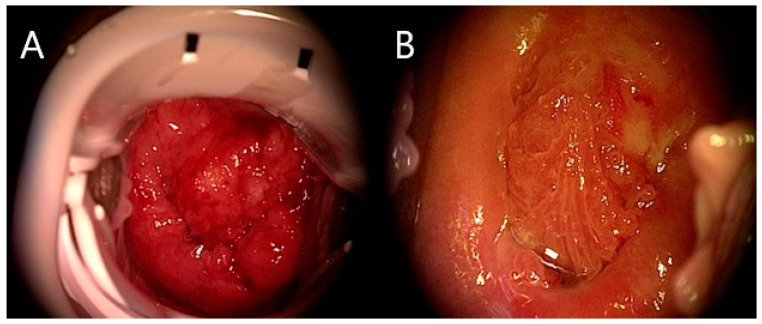
Cervical cancer seen during colposcopic investigation before (**A**) and after (**B**) chemotherapy.

Due to the fact that no pelvic metastases were reported, the standard treatment with radical hysterectomy was suggested. However, the patient refused this option, due to the fact that fertility preservation was strongly desired. As a result, 10 days after diagnostic laparoscopy neoadjuvant chemotherapy had been started and 3 cycles of carboplatin/paclitaxel were carried out every 3 weeks, excellent treatment response to the chemotherapy was achieved, with a considerable decrease in tumor size. Figure 2 visualizes the uterine cervix before and after the chemotherapy.

One month after the third chemotherapy cycle, laparoscopic radical trachelectomy (without uterine manipulator) was conducted. The procedure is shown in Video S3. Histopathological examination revealed invasive G3 cervical cancer; according to T criteria, it was ypT1a1. Stromal invasion was estimated to be 2 mm in depth and 3 mm in width, and specimen margins were all negative.

**Figure 3:**
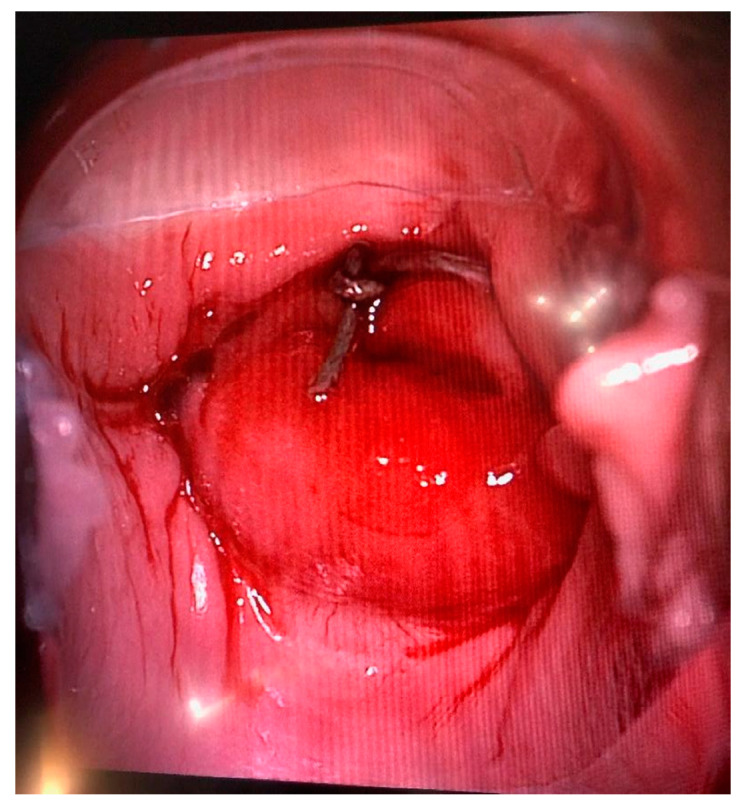
Colposcopy performed 2 months after radical trachelectomy.

Colposcopy with cervical stump biopsy was performed 2 months after radical trachelectomy, obtaining four specimens of up to 0.3 cm in diameter (Figure 3). There were no pathological changes in all specimens investigated.

The patient had undergone Pap smear 6 months after trachelectomy—neither intraepithelial lesion, nor HPV16 infection was found, and the CINtec PLUS cytology test was also negative. Colposcopy and Pap smear were repeated 10 months after trachelectomy, showing the same results as reported above.

The patient, 27-years-old, proceeded to the in vitro fertilization (IVF) procedure 14 months after radical treatment. Endometrial scratching and ovarian puncture were performed, and two eggs were collected. They were fertilized with intracytoplasmic sperm injection (ICSI), and one embryo was inserted into the uterine cavity. Unfortunately, the IVF procedure was unsuccessful at that time. The next IVF procedure is planned in the near future.

## 3. Discussion

Several data suggested that, for patients with early-stage CC, safety and clinical outcomes were equal for radical trachelectomy and RH (in terms of operative time, blood loss, post-operative complications, recurrence rate, and 5-years OS) [2].

Currently, a patient’s suitability for the radical trachelectomy is determined by the size of the primary lesion and whether the oncological process has spread to lymph nodes and distant organs. This assessment is performed using colposcopy, MRI, or PET [5]. In addition, proper histological assessment is crucial [1]. Radical trachelectomy is recommended to be avoided in case of lesion extensions of more than 1 cm into the isthmus. The existing evidence implies that patients who are at high-risk for relapse significantly more often have adenocarcinoma, neuroendocrine tumor, gastric-type adenocarcinoma (therefore, these cases should be excluded from radical trachelectomy), and lymph-vascular space invasion (LVSI), and they frequently undergo lymph node dissection (LND) prior to neoadjuvant therapy, showing an inadequate response to it [1].

FSS for CC treatment includes traditional cone biopsy, cervical amputation, and radical trachelectomy, which is under the interest of our report. Radical trachelectomy may be performed by vaginal, abdominal, or minimally invasive (as in our case) approaches [3]. Based on the available data, the oncological results in patients having stage IB1 disease are quite similar, despite the procedure applied. A great debate within the academic arena was evoked by the Laparoscopic Approach to Cervical Cancer (LACC) research study, giving rise to doubt regarding the oncological outcomes of the laparoscopic radical trachelectomy approach for the early-stage CC. Most of the patients undergoing FSS have had tumor sizes below 2 cm in diameter, while only 47% of the LACC women had tumors less than 2 cm in size. Hence, it is unknown if this data could be applicable for a minimally invasive surgery (MIS) approach for radical trachelectomy [6].

Based on the current knowledge regarding the oncologic outcomes after MIS approach radical trachelectomy, the recurrence rate after laparoscopic radical trachelectomy is 11%, highlighting that, in 40% of these cases, the tumor were sized above 2 cm in diameter. Another MIS-RT study, although with a limited number of patients, reported a combined recurrence rate of 4.2% and rate of deaths below 1%, which made the outcomes similar to open and vaginal approaches [6]. Other authors reported that outcomes following distinct types of trachelectomy suggested that patients undergoing MIS experienced slightly higher recurrence rates and cervical suture erosion, as well as extreme premature delivery rates, although they benefited from a higher chance of achieving pregnancy and live birth, with lower rates of cervical stenosis [3]. Meta-analysis, which included 53 studies, reporting recurrences with a median follow-up of 12 months in early CC (FIGO 2009 IA with LVSI, IB, and IIA) after fertility sparing treatment, concluded that all approaches of trachelectomy had corresponding oncologic outcomes, while the best obstetrics sequelae was achieved after the vaginal approach [6]. Although the follow-up was too short, we believe that this recent meta-analysis provides systemized evidence from a considerable number of studies. Others argued that MIS was more related to vaginal approach pregnancy outcomes and acted favorably [7]. Fertility outcomes after the MIS approach were similar to other radical trachelectomy approaches, in terms of pregnancy rate and live birth rate, at 36.2% and 57.1%, respectively [6]. The most considerable controversy is the size of the tumor, as oncological outcomes vary widely among authors. For instance, the National Comprehensive Cancer Network (NCCN) does not strictly exclude patients with tumors greater than 2 cm for trachelectomy, while the European guidelines state that fertility sparing surgery in lesions > 2 cm is an experimental approach [1,3].

Some authors evaluated fertility and oncological outcomes after neoadjuvant chemotherapy (NACT), followed by fertility sparing surgery (VRT, ART, LRT) for CC 2–4 cm in size. Meta-analysis, reporting outcomes of 114 patients, showed that, out of 64 pregnancy efforts, there were 49 (76.6%) live births (6 (9.4%) preterm deliveries). Concerning oncological outcomes, 6.1% of patients had relapse, and two patients died of the disease. This study concludes that fertility sparing surgery after NACT is a promising option for CC patients with tumors 2–4 cm in size who wish to conceive [8]. The National Cancer Database retrospective analysis revealed a similar survival rate for 246 patients who underwent radical trachelectomy for stages IA2–IB (FIGO 2009) CC either by MIS (58.5%) or by abdominal (41.5%) approach. During the follow-up (37 months for the MIS group and 40 months for the laparotomy group), there were a total of 11 deaths (5.3%), 4 (3.5%) in the MIS group and 7 (7.6%) in the laparotomy group, showing no statistical significance [9].

Up until now, nearly 225 cases (39 cases of tumors measuring 2–4 cm) of LRT without NACT have been reported. Disease recurrence was observed in 13 patients (5.7%), and 8 of them have had tumors 2–4 cm in size. These results were comparable to tumors >2 cm treated by VRT [10]. Other authors, comparing ART, VRT, and NACT, followed by radical trachelectomy approaches for tumors size 2–4 cm, reported that the highest fertility preservation rate was achieved in the VRT group, and the highest pregnancy rate was achieved in the NACT group, followed by radical trachelectomy group, while highest recurrence rate was achieved in the VRT group for tumors above 2 cm in diameter, discouraging us to choose this approach for tumors >2 cm, despite positive fertility results [10].

The fertility and oncologic outcomes of women affected by CC >4 cm (FIGO IB3) who received NACT (platinum-based) and FSS were assessed by systematic review. The 4.5-year DFS was 92.3%, and all the patients survived. Recurrence occurred in two patients who received ifosfamide, instead of paclitaxel, and had residual disease found during the histopathological investigation. Four out of six patients achieved at least one pregnancy, and three out of five showed preterm delivery [11].

At present, there is growing number of evidence of the role of NACT, with a significant reduction of primary tumors seeking safe and reasonable fertility sparing surgery [1,6,12]. A review article by Kuznicki et al. showed that response rate to NACT varies from 84% to 100%, although complete responses were seen only in 21–44% of the cases [6]. The systematic review data described the results of NACT, followed by a VRT in the patients with the stage IB2 disease. The total recurrence and death rates were comparable to ART and resulted in 10% and 2.9%, respectively. It is worth mentioning that the benefits of the NACT and VRT strategies were higher pregnancy and live births rates, compared to ART [13]. Total pregnancy rate was 70% among women who tried to conceive, and 63% of these pregnancies were successfully followed by live births. According to the results of mentioned studies, NACT and less radical surgical treatments are feasible and effective methods for increasing successful pregnancy rates in CC patients.

According to recent studies, the relapse rates after NACT following FSS vary from 6% to 10% for tumors below 2 cm in diameter [7,13,14]. Unfortunately, the relapse rate for tumors more than 2 cm in diameter increased up to 12.8% [15]. The relapse rates after ART without NACT vary from 6.9% to 7% [7,13]. The pregnancy rate tends to be higher in NACT following FSS strategy—it varies from 25.7% to 70%, with live births rates of 63–76.6%. Meanwhile, the rates of pregnancy and live births after ART are 21% and 42%, respectively [13,14]. Patients undergoing ART tend to have better oncologic control of the disease.

Recent review by Buda et al. altogether analyzed 114 women with stage IB2 cervical cancer who underwent NACT and FSS, showing that only in 76.7% of cases uterine preservation was achieved [16]. The best response rate was reported with the cisplatin, ifosfamide, and paclitaxel regime, and the optimal response was recorded in 60.9% of cases. Total recurrence rate was 9.2%, median DFS time was 7.4 months, and death rate was 4.6%. Half of the patients who tried to conceive and had undergone NACT prior to conization appeared to have the most favorable obstetrics outcomes [16]. The ongoing CONTESSA trial, which includes patients with stage IB2 cervical cancer receiving NACT before a conservative surgery, will provide stronger evidence on this topic [16,17].

IVF is sometimes recommended after radical trachelectomy because of cervical stenosis and other causes of natural pregnancy difficulties. In addition, it was reported that radical trachelectomy may diminish ovarian reserve, since a decreased response to controlled ovarian stimulation had been reported after radical trachelectomy procedures [18]. On top of that, patients who conceived after FSS had a higher chance of preterm delivery between 32 and 37 gestation weeks, as well as higher neonatal morbidity risk, compared to the controls [19].

## 4. Conclusions

This case report and literature review revealed that FSS approaches and perioperative management in the case of CC needs further research, in order to achieve both the satisfying oncologic outcomes and desired obstetrics results. This strongly applies to the role of NACT seeking tumor reduction before performing less radical surgery and the safety of FSS for tumors above 2 cm in diameter. Currently, there is a lack of strict algorithms on radical trachelectomy approaches, and the choice is usually achieved individually, depending on the experience of the surgeon.

## Data Availability

Not applicable.

## Supplementary Materials

The following supporting information can be downloaded at: https://www.mdpi.com/article/10.3390/medicina58121827/s1, Video S1. Colposcopic image of cervix during initial evaluation. Video S2. Indocyanine green (ICG)—enhanced fluorescence—guided laparoscopy. Video S3. Laparoscopic radical trachelectomy.

## Abbreviations

ART—abdominal radical trachelectomy; ASCUS—atypical squamous cells of undetermined significance; CC—cervical cancer; CT—computerized tomography; DFS—disease-free survival; ESGO—European Society of Gynaecological Oncology; FSS—fertility-sparing surgery; HPV—human papillomavirus; HSIL—high-grade squamous intraepithelial lesion; ICG—indocyanine green; ICSI—intracytoplasmic sperm injection; IVF—in vitro fertilization; LACC—laparoscopic approach to cervical cancer study; LND—lymph node dissection; LRT—laparoscopic radical trachelectomy; LVSI—lymph-vascular space invasion; MIS—minimally invasive surgery; MRI—magnetic resonance imaging; NACT—neoadjuvant chemotherapy; NCCN—National Comprehensive Cancer Network; PET—positron emission tomography; PLND—pelvic lymph node dissection; RH—radical hysterectomy; SN—sentinel node; VRT—vaginal radical trachelectomy.
